# Prevalence of sacral dysmorphia in a prospective trauma population: Implications for a "safe" surgical corridor for sacro-iliac screw placement

**DOI:** 10.1186/1754-9493-5-8

**Published:** 2011-05-10

**Authors:** Erik A Hasenboehler, Philip F Stahel, Allison Williams, Wade R Smith, Justin T Newman, David L Symonds, Steven J Morgan

**Affiliations:** 1 Department of Orthopaedic Surgery, Denver Health Medical Center, University of Colorado Denver, School of Medicine, 777 Bannock Street, Denver, CO 80204, USA; 2 Eastern Colorado Health Care System, Denver Veterans' Affairs Medical Center, Denver, CO 80220, USA; 3 Department of Orthopaedic Surgery, Geisinger Medical Center, 100 North Academy Ave, Danville, PA 17825, USA; 4 Department of Radiology, Denver Health Medical Center, University of Colorado School of Medicine, 777 Bannock Street, Denver, CO 80204, USA

## Abstract

**Background:**

Percutaneous sacro-iliac (SI) screw fixation represents a widely used technique in the management of unstable posterior pelvic ring injuries and sacral fractures. The misplacement of SI-screws under fluoroscopic guidance represents a critical complication for these patients. This study was designed to determine the prevalence of sacral dysmorphia and the radiographic anatomy of surgical S1 and S2 corridors in a representative trauma population.

**Methods:**

Prospective observational cohort study on a consecutive series of 344 skeletally mature trauma patients of both genders enrolled between January 1, 2007, to September 30, 2007, at a single academic level 1 trauma center. Inclusion criteria included a pelvic CT scan as part of the initial diagnostic trauma work-up. The prevalence of sacral dysmorphia was determined by plain radiographic pelvic films and CT scan analysis. The anatomy of sacral corridors was analyzed on 3 mm reconstruction sections derived from multislice CT scan, in the axial, coronal, and sagittal plane. "Safe" potential surgical corridors at S1 and S2 were calculated based on these measurements.

**Results:**

Radiographic evidence of sacral dysmorphia was detected in 49 patients (14.5%). The prevalence of sacral dysmorphia was not significantly different between male and female patients (12.2% *vs*. 19.2%; *P *= 0.069). In contrast, significant gender-related differences were detected with regard to radiographic analysis of surgical corridors for SI-screw placement, with female trauma patients (*n *= 99) having significantly narrower corridors at S1 and S2 in all evaluated planes (axial, coronal, sagittal), compared to male counterparts (*n *= 245; *P *< 0.01). In addition, the mean S2 body height was higher in dysmorphic compared to normal sacra, albeit without statistical significance (*P *= 0.06), implying S2 as a safe surgical corridor of choice in patients with sacral dysmorphia.

**Conclusions:**

These findings emphasize a high prevalence of sacral dysmorphia in a representative trauma population and imply a higher risk of SI-screw misplacement in female patients. Preoperative planning for percutaneous SI-screw fixation for unstable pelvic and sacral fractures must include a detailed CT scan analysis to determine the safety of surgical corridors.

## Introduction

Percutaneous sacro-iliac (SI) screw fixation represents an established standard and widely used technique in the management of unstable posterior pelvic ring injuries and sacral fractures [1-4]. The misplacement of percutaneous SI-screws represents a critical complication which occurs in about 10% to 15% of all cases, despite apparent accuracy on intraoperative fluoroscopy [5-8]. In this regard, fracture malreduction and preexisting sacral deformities have been recognized as risk factors for inadequate surgical corridors with an increased incidence of SI-screw misplacement and the potential for neurovascular complications [9-12]. More recently, CT-guided techniques were described to reduce the inherent risk of an unperceived SI-screw misplacement by pure reliance on intraoperative fluoroscopic guidance [13-15]. Other authors recommended the use of intraoperative neurophysiological monitoring during the placement of iliosacral screws [16].

Several groups have previously investigated the anatomy of the sacral corridor for SI-screw placement by radiographic or cadaveric studies, in order to increase surgical accuracy and to decrease the risk of intraoperative complications [9,12,17-28]. However, up to date, limited information is available on the 3-dimensional anatomy of the sacrum and its relation to a "safe" SI-screw placement [12,29]. The present study was designed to assess the radiographic 3-dimensional surgical corridor at S1 and S2 and to determine the prevalence of a dysmorphic sacrum in a representative prospective trauma population.

## Patients and methods

This study was approved by the institutional review board (No. 06-0789). All consecutive trauma patients between 18 years and 70 years of age admitted for to our institution between January 1, 2007 and September 30, 2007, who underwent a pelvic supine computed tomography (pCT) scan as part of their initial trauma work up, were prospectively included in the study. No CT adjustments were made to possible anatomical variations of the lumbo-sacral level. Exclusion criteria consisted of age <18 or >70 years, presence of a sacral tumor or infection, sacral agenesis, previously fixated sacral fracture and CT scans with malpositioned patients. In addition, patients with bilateral sacral fractures were excluded from this study due to the inability of assessing normal sacral anatomy. A power analysis was performed prior to initiation of the study, with α set at 0.05 and power at 0.8, which determined a sample size of 344 subjects required of either gender, based on twelve different measured variables on pCT scans. The analysis of pCT sections was performed on 3 mm reconstruction sections derived from multislice CT scan (GE Light Speed Plus, GE Medical Systems, Chalfont St. Giles, UK) on a GE Advantage Workstation using 4.3_05 Software, volume viewer 2 (GE Medical Systems). For determination of 3-dimensional sacral morphology, pCT sections were assessed on axial, coronal, and sagittal reconstructions.

**Figure 1:**
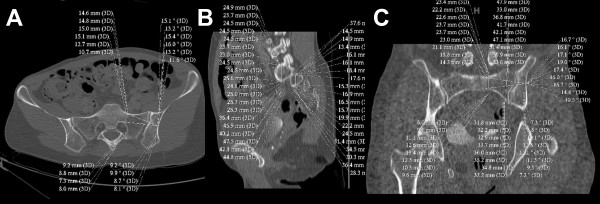
**Technique and obtained CT measurements summary in the axial (panel A), sagittal (panel B), and coronal planes (panel C)**. See text for details and explanations.

In the axial plane (Figure 1A), measurements consisted of the distance between the anterior cortex of the sacral ala to the posterior wall of the S1 and S2 foramina, and the "surgical angle". The mean of all measurements for S1 and S2 was used to determine the "safe axial surgical corridor" at its narrowest point. The axial surgical angle was determined by selecting the ideal entry point on the ilium. A perpendicular line was drawn to the midpoint of the widest axial surgical canal view and extended out through the ilium. The intersection of this line with the ilium was used as the apex for angle calculation. The mean value of all angles was used to determine the "safe axial surgical angle" for S1 and S2, respectively.

In the sagittal plane (Figure 1B), the height of S1 and S2 bodies was measured by drawing a perpendicular line to the midpoint of both sacral bodies. The mean of these values was used to define the "safe surgical sagittal corridor". No angle was measured in the sagittal plane.

In the coronal plane (Figure 1C), the tallest and smallest sacral body height for S1 and S2 were measured, and the mean coronal body height was calculated from these values. As for the axial view, a "safe surgical angle" was also measured in the coronal plane. A perpendicular line was drawn first to the midpoint of the most narrow S1 and S2 body height in its widest view on pCT slices. The intersection of this line with the ilium was then used as the apex to measure the safe surgical angle, with a mean value used to define the "safe surgical coronal angle".

In addition, sacral morphology was independently evaluated for each patient by three fellowship-trained orthopaedic trauma surgeons (PFS, WRS, SJM) who were blinded about patient identity and the measured values on pCT sections. The surgeons were asked to evaluate sacral anatomy from each patient as either "normal" or "dysmorphic". The definition of "sacral dysmorphia" has not been unequivocally defined in the literature. For the present study, we defined the presence of sacral dysmorphia to include any of the following criteria: Altered sacral anatomy with an increased alar slope, obliquity of the residual transverse process on the sacral ala, anomaly of the first sacral anterior neural foramina anatomy, and sacralized L5 or lumbarized S1 vertebrae, as independently assessed by the three attending surgeons, and agreed upon in a consensus discussion evaluating all potentially equivocal cases.

In addition, demographic data and injury characteristics were recorded for all patients, including age, gender, ethnicity, mechanism of injury, and fracture classification using the AO/OTA, Young and Burgess, and Denis classification systems.

All sacral measurements derived from pCT sections were recorded in a computerized database (SPSS^® ^11.5, Chicago, IL). Univariate statistical analysis was performed for each measured value derived from pCT analyses, for demographic variables, injury characteristics, and for the prevalence of sacral dysmorphia. After determining distribution of data (parametric *vs*. non-parametric), the Student's *t*-test and Mann-Whitney-*U *test, respectively, were used to determine differences in sacral morphology between genders. The Bonferroni correction was applied to account for multiple interindividual comparisons. Data were considered statistically significant at *P *< 0.05.

## Results

A total of 344 consecutive patients (245 males, 99 females) were prospectively included in this study. The mean age was 36.9 years in males and 36.8 years in females. Injury mechanisms include motor-vehicle accidents (*n *= 157), pedestrian versus automobile (*n *= 37), fall from height (*n *= 67), assaults (*n *= 30), motorcycle accidents (*n *= 27), crush injuries (*n *= 8), bicycle versus automobile (*n *= 7), stab wounds (*n *= 5), gunshot wounds (*n *= 4) and other mechanisms (*n *= 2). Secondary radiographic findings on pCT analysis include degenerative L5/S1 osteochondrosis (*n *= 42), sacralized L5 vetrebral body (*n *= 28), and degenerative SI joint arthritis (*n *= 21).

Significant differences between genders were found for all measurements, with female patients having significantly lower values than males in all pCT sections and 3-dimensional planes assessed. As shown in table 1, these measurements consisted of the axial S1 and S2 foramen-anterior cortex (S1FAC, S2FAC), the tallest coronal S1 and S2 body height (TS1BHT, TS2BHT), the sagittal S1 and S2 mean body height (S1SAG.mean, S2SAG.mean), the axial S1 and S2 angle (Angle1A), and the coronal S1 angle (Angle1C).

**Table 1:** Mean variables (± SD) measured on pelvic CT sections in the prospective trauma cohort (*n *= 344), stratified by gender

Variable	Male patients (*n *= 245)	Female patients (*n *= 99)	Statistical test applied	*P*-value
Age	36.9 ± 12.98	36.8 ± 14.71	Mann-Whitney-*U *	0.584

S1FAC	17.85 ± 2.71	16.18 ± 2.21	Student's *t*-test	**<0.001**

S2FAC	11.83 ± 2.34	10.78 ± 2.09	Student's *t*-test	**<0.001**

TS1BHT	46.74 ± 9.12	43.10 ± 8.87	Mann-Whitney-*U *	**<0.001**

SS1BHT	23.19 ± 3.91	21.17 ± 3.41	Mann-Whitney-*U *	**<0.001**

S1SAG.mean	31.39 ± 3.56	28.97 ± 4.11	Mann-Whitney-*U *	**<0.001**

TS2BHT	32.82 ± 6.96	29.73 ± 5.71	Mann-Whitney-*U *	**<0.001**

SS2BHT	16.10 ± 3.63	15.13 ± 3.80	Mann-Whitney-*U *	**<0.001**

S2SAG.mean	25.48 ± 3.94	23.79 ± 3.68	Mann-Whitney-*U *	**<0.001**

ANGLE1A	20.07 ± 3.21	17.31 ± 2.80	Student's *t*-test	**<0.001**

ANGLE1C	19.38 ± 3.28	18.23 ± 4.26	Mann-Whitney-*U *	**<0.001**

ANGLE2A	13.56 ± 2.97	11.97 ± 2.37	Mann-Whitney-*U *	**<0.001**

ANGLE2C	16.13 ± 2.88	14.21 ± 2.73	Student's *t*-test	**<0.001**

Statistically significant differences were found for all measurements in all 3-dimensional planes between genders, with female patients having significantly reduced surgical S1 and S2 corridors than male counterparts. See text for abbreviations and explanations.

Abbreviations: S1FAC and S2FAC = S1 and S2 foramen-anterior cortex

TS1BHT and T2SBHT = tallest coronal S1 and S2 body height/the sagittal S1 and S2 mean body height

SS1BHT and SS2BHT = smallest S1 and S2 body height

S1SAG.mean and S2SAG.mean = S1 and S2 sagittal mean height

Angle 1A/2A and 1C/2C = axial S1 and S2 angle and coronal S1/S2 angle

Sacral dysmorphia was presented in 49 of 344 patients (14.2%). Female patients had a higher prevalence of dysmorphic sacral morphology (19.2%) than male patients (12.2%), however, the difference was not statistically significant (p = 0.069).

**Figure 2:**
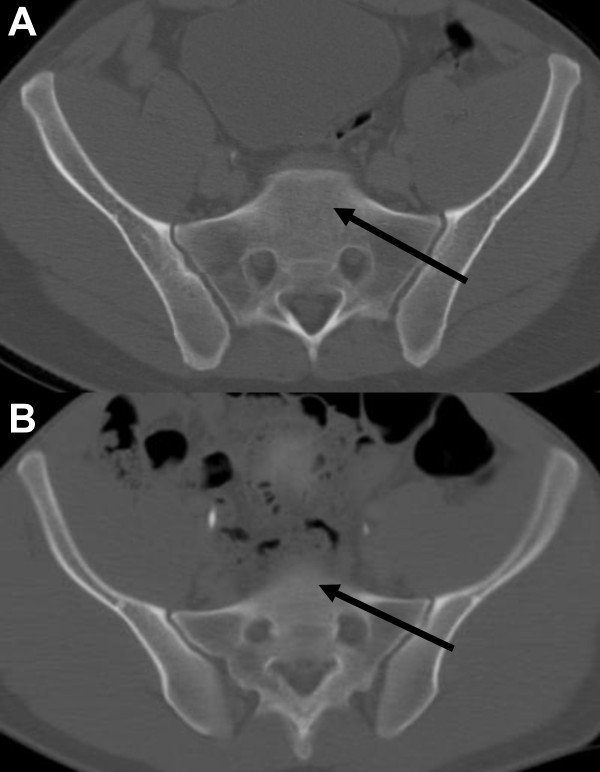
**Example of a "safe surgical corridor" for SI-screw trajectories in a normal (panel A) and dysmorphic sacrum (panel B)**. Based on the vestibular concept described by Carlson et al. [29], the entry point was placed perpendicular to the narrowest point at the level of the sacral foramen, the so-called "vestibule". See text for details and explanations.

The pCT measurements were furthermore compared between the groups with "normal" (*n *= 295) versus "dysmorphic" (*n *= 49) sacral morphology (table 2). All measurements on the S1 vertebral body, with according corridors and angles, were significantly diminished in the sacral dysmorphia group, compared to the cohort with normal sacral morphology (table 2). A case example depicting the anatomic variation with a steeper first alar slope and narrower surgical canal in a patient with sacral dysmorphia is shown in Figure 2.

**Table 2:** Mean variables (± SD) measured on pelvic CT sections in the prospective trauma cohort (*n *= 344), stratified by sacral morphology.

Variable	Normal sacral morphology (*n *= 295)	Dysmorphic sacral morphology (*n *= 49)	Statistical test applied	*P*-value
Age	36.50 ± 13.35	39.35 ± 14.13	Student's *t*-test	0.171

S1FAC	17.69 ± 2.50	15.43 ± 2.93	Student's *t*-test	**<0.001**

S2FAC	11.45 ± 2.28	12.01 ± 2.49	Student's *t*-test	0.117

TS1BHT	46.93 ± 7.55	38.29 ± 13.73	Student's *t*-test	**<0.001**

SS1BHT	22.84 ± 3.35	21.21 ± 6.02	Mann-Whitney-*U *	0.028

S1SAG.mean	31.02 ± 3.54	28.69 ± 5.09	Mann-Whitney-*U *	**0.001**

TS2BHT	31.53 ± 6.62	34.36 ± 7.19	Student's *t*-test	0.06

SS2BHT	15.71 ± 3.74	16.45 ± 3.40	Mann-Whitney-*U *	0.078

S2SAG.mean	24.97 ± 3.72	25.12 ± 5.12	Student's *t*-test	0.817

ANGLE1A	19.65 ± 3.19	17.02 ± 3.37	Student's *t*-test	**<0.001**

ANGLE1C	19.33 ± 3.34	17.38 ± 4.70	Mann-Whitney-*U *	**0.003**

ANGLE2A	13.10 ± 2.94	13.13 ± 2.67	Mann-Whitney-*U *	0.811

ANGLE2C	15.50 ± 2.92	16.02 ± 3.18	Student's *t*-test	0.258

Statistically significant differences were found for all measurements in all 3-dimensional planes between patients with normal versus dysmorphic sacral morphology at the S1 level. In contrast, differences at S2 were not statistically significant. Patients with sacral dysmorphia showed a trend towards increased mean S2 vertebral body heights, implying the S2 route as a "safe" alternative for placement of SI = screws in presence of sacral dysmorphia. See table 1 for abbreviations.

Abbreviations: S1FAC and S2FAC = S1 and S2 foramen-anterior cortex

TS1BHT and T2SBHT = tallest coronal S1 and S2 body height/the sagittal S1 and S2 mean body height

SS1BHT and SS2BHT = smallest S1 and S2 body height

S1SAG.mean and S2SAG.mean = S1 and S2 sagittal mean height

Angle 1A/2A and 1C/2C = axial S1 and S2 angle and coronal S1/S2 angle

In contrast, no statistically significant difference was detected for any measurement or angle related to the S2 vertebral body between patients with normal versus dysmorphic sacral morphology (table 2). Strikingly, the differences in tallest S2 body height (TS2BHT) showed an inverse relation, with increase values in the dysmorphic group (34.36 ± 7.19), compared to the cohort with normal sacral morphology (31.53 ± 6.62). While these data just approached statistical significance (*P *= 0.06), the finding implies a compensatory increase of S2 body height in the population with dysmorphic sacra (table 2).

Based on the vestibular concept described by Carlson et al. [29], we determined the "ideal" SI-screw corridor as perpendicular to the "vestibule", which is defined as the most narrow "safe zone" through which an SI-screw would pass at S1 and S2 (Figure 2). Based on our measurements, an oblique trajectory of SI-screws within the "vestibule" will significantly diminish the "safe zone" and increase the potential risk of inadvertent screw misplacement. In contrast, the strict perpendicular vestibular trajectory will allow the safe placement of SI-screws in all patients with sacral dysmorphia analyzed in the present study, as illustrated by a case example in Figure 2, panel B.

## Discussion

This prospective observational cohort study demonstrates a high prevalence of sacral dysmorphia of 14.2% in a representative trauma population. A trend was observed for a higher prevalence in females (19.2%) compared to male subjects (12.2.%), which, however, was not statistically significant (*P *= 0.069). A significant difference between genders was detected by analysis of pCT sections in all 3-dimensional planes (axial, sagittal, coronal), for both S1 and S2 vertebral bodies. The female cohort had significantly decreased measurements and angles than male counterparts, implying decreased "safe" surgical corridors for potential SI-screw placement in women. In addition, the pCT analysis revealed significantly decreased corridors at S1, but not S2, between the groups with "normal" versus "dysmorphic" sacral morphology. In contrast, S2 vertebral body height was increased in patients with sacral dysmorphia, albeit without reaching statistical significance (*P *= 0.06).

Since the first description, the technique of fluoroscopy-guided percutaneous SI-screw fixation of unstable posterior pelvic ring injuries and sacral fractures has seen a worldwide dissemination and is currently considered a standard of care [1-3]. Despite the frequent application and the advantages of this minimally invasive technique, percutaneous SI-screw remains inherently associated with the risk of a significant neurovascular complication due to inadvertent screw misplacement [7,10,11]. The rate of SI-screw misplacement in the pertinent literature is around 10% to 15% [5-8]. This rate may just represent the "tip of the iceberg", since many surgeons do not obtain standardized postoperative CT scans for quality control.

Multiple radiographic and cadaveric studies have been previously published on the sacral anatomy and its relation to "safe" surgical corridors for SI-screw placement [9,17,18,21-23,25,26,28-32]. However, only few studies have addressed the 3-dimensonal "surgical corridor" for SI-screw placement [12,29]. In addition, to our knowledge, no study has been conducted until present which analyzed the prevalence of sacral dysmorphia and its relevance for a "safe" surgical corridor in a representative trauma population. In the present study, we report the impressive prevalence of 14.5% of sacral dysmorphia among general trauma patients. Furthermore, we describe a significantly compromised 3-dimensional surgical corridor at the S1 level in patients with sacral dysmorphia, compared to the group with normal sacral morphology. Finally, we found that trauma patients of female gender have a significantly narrower 3-dimensional "safe" surgical corridor at S1 and S2, compared to male patients.

Routt *et al*. have previously compared the radiographic anatomy of eighty patients with complex pelvic fractures treated by percutaneous SI-screw fixation, with the pelvic anatomy of 10 cadaveric dissections [9]. The authors found a high incidence of surgically important abnormal morphological patterns of sacral anatomy, which were identified by pelvic outlet and lateral sacral plain radiographs combined with computed tomographic scans [9]. Similar to those results, we found anatomic variations with a steeper first alar slope and narrower surgical canal in patients with sacral dysmorphia.

An article by Carlson and colleagues described the "vestibule" concept for safe placement of S1 and S2 screws by multiplanar 2-dimensional CT reformatting in 30 consecutive normal subjects who consented to have a pelvic CT scan performed [29]. Hereby, the "vestibule" was defined as the most narrow "safe zone" through which an SI-screw would pass at S1 and S2. The findings from our present study on a much larger cohort of prospective trauma patients (*n *= 344) concur with the suggested vestibular concept described by Carlson and colleagues [29]. In the present study, we invariably found the "safe" surgical corridor to be located perpendicular to the center of the narrowest point ("vestibule") at the level of the sacral foramen, both for normal (Figure 2A) and dysmorphic sacral morphology (Figure 2B).

Carlson *et al*. further described a new classification of sacral morphology [29] which has not yet been further validated by additional prospective studies. Sacral anatomy was classified into a type 1 "capacious" sacrum (>1.2 cm), a more narrow type 2 "intermediate" sacrum (<1.2 cm, >0.75 cm) and a type 3 "dysplastic" sacrum (<0.75 cm), depending on the measured vestibular corridor. In our representative cohort of 344 consecutive prospective patients, we determined a sacral morphology as either type 1 or type 2, with a mean axial corridor of 1.73 cm (S1) and 1.15 cm (S2), respectively. Despite the finding of 49 patient with sacral dysmorphia in our study, we did not detect a single patient with a type 3 "dysplastic" sacrum (<0.75 cm) according to the criteria by Carlson and colleagues [29]. The measured angles in the axial and coronal view, for both S1 and S2 corridors, furthermore revealed a "safe zone" for SI-screw placement if inserted through in the middle of the vestibule (Figure 2). The mean axial S1 and S2 angle for our population was 19.27° and 13.10° degrees, respectively.

In contrast to the study by Carlson et al., we selected one single entry point, for either axial or coronal planes (Figure 1A, C), at the level of the pCT section with the widest corridor, which was then used as the basis for all radiographic measurements. No surgical angles and corridors could be calculated from the sagittal plane, which served for calculation of the mean S1 and S2 sacral body heights measured from the alar slope across the sacral foramen (Figure 1B). Based on our current data, we determined the "ideal" SI-screw corridor as perpendicular, not oblique, to the "vestibule", which is of crucial importance particularly in dysmorphic sacra (Figure 2). Based on our measurements, the oblique placement of SI-screws within the "vestibule" will significantly diminish the "safe zone" and increase the risk of inadvertent screw misplacement with possible neurovascular complication. In contrast, the strict perpendicular vestibular placement will allow the safe placement of SI-screws in any patient with sacral dysmorphia (Figure 2B).

Another interesting finding in our study is the detection of higher mean S2 body heights in patients with dysmorphic sacra, compared to patients with normal sacral anatomy (table 2). This finding implies that S2 may represent the "safe surgical corridor" of choice in patients with sacral dysmorphia. This notion is supported by a recent paper by Moed and Geer who recommended SI-screw fixation at S2 as a safe and effective technique in 49 patients with unstable posterior pelvic ring disruptions [33]. However, due to the irregular and smaller width at the different individual S2 corridor levels determined in the present study, we recommend a careful analysis of all pCT sections in all 3-dimensional views, in order to avoid an inadvertent SI-screw misplacement.

There are limitations to this study. This is the first study of its kind to evaluate the incidence of sacral dysmorphia and its possible implication on the surgical corridor in a large trauma population. Although our measurements are easily reproducible with any multislice CT scanner used, not every institution offers the availability of thin-cut CT slices of 2.5 mm to 3 mm, and the required software for obtaining accurate measurements. The pre-hoc power analysis of our study determined a cohort of 344 patients needed for statistical significance. However, based on the established prevalence of sacral dysmorphia in this study (14.5%), from an epidemiological perspective, at least twice-fold increased overall sample numbers (*n *= 788) would be required to determine significant gender-associated differences in the prevalence of sacral dysmorphia. Thus, our study was theoretically underpowered to determine differences in the prevalence of sacral dysmorphia between male and female patients, implying a potential type I error. Finally, another drawback of our present study is the fact that the radiographic measurements were made on non-injured pelvic and sacral CT scans. Thus, the finding that any sacral morphology, including dysmorphic sacra, would still allow the potential "safe" placement of SI-screws with a trajectory perpendicular to the "vestibule" (Figure 2B), will obviously depend on a perfect anatomic reduction of SI-joint dislocations prior to fixation. Reilly and colleagues have previously reported a substantial compromise of available SI-screw space in malreduced sacral fractures and fracture-dislocations with a residual displacement >1 cm [12]. Our present findings would certainly concur with those data, emphasizing the "classic" notion of a perfect anatomic reduction as a "condition-sine-qua-non" for successful management strategies in orthopaedic trauma.

In conclusion, our data imply a high prevalence of sacral dysmorphia (14.5%) in a prospective, representative trauma population with an increased risk of SI-screw misplacement at S1. Adherence to the "vestibular concept" with perpendicular screw placement at the most narrow corridor would still allow a "safe" S1 screw placement in dysmorphic sacra, with the prerequisite of a perfect, anatomic SI-joint reduction. Based on the finding of increased S2 body heights in patients with sacral dysmorphia, the S2 trajectory may represent a "safe" alternative for SI-screw placement in those patients. Finally, female gender was associated with significantly reduced S1 and S2 corridors in all planes, compared to male counterparts, implying a "high level of suspicion" for the potential risk of SI-screw misplacement in female patients. As in any fracture case, preoperative planning is imperative. A meticulous analysis of pelvic CT scans in all its three dimensions will allow estimating the average size of the "vestibule" in order to determine the ideal entry point and screw orientation. Only those trained and experienced in their use should perform percutaneous SI-screw fixation.

## Competing interests

The authors declare that they have no competing interests.

## Authors' contributions

PFS and WRS designed this study. EAH, JTN, and DLS performed all measurements of sacral corridors in three planes. PFS, WRS and SJM assessed all pelvic X-rays for presence of sacral dysmorphia. AEW performed the statistical analysis of the data. EAH wrote the first draft of this manuscript. PFS revised the manuscript. All authors read and approved the final version of this paper.
